# A case report of extranodal NK/T cell lymphoma nasal-type with direct pulmonary involvement

**DOI:** 10.1097/MD.0000000000044411

**Published:** 2025-09-19

**Authors:** Hui Zhang, Jiaming Yin, Haibo Ge

**Affiliations:** a Pulmonary and Critical Care Medicine, Nanjing Hospital of Chinese Medicine Affiliated to Nanjing University of Chinese Medicine, Nanjing, Jiangsu Province, China; b Nanjing University of Chinese Medicine, Nanjing, Jiangsu Province, China.

**Keywords:** EB virus infection, extranodal NK/T-cell lymphoma, hematological neoplasm

## Abstract

**Rationale:**

Extranodal nasal-type NK/T-cell lymphoma primarily invades the nasal and nasopharyngeal regions, while direct pulmonary involvement is extremely rare and prone to clinical misdiagnosis or oversight.

**Patient concerns:**

A 45-year-old man presented with recurrent high fever and cough. Anti-infective therapy and the peaspartase + methotrexate + etoposide + dexamethasone regimen proved ineffective, with chest CT demonstrating disease progression.

**Diagnoses:**

A patient with extranodal nasal-type NK/T-cell lymphoma, classified as stage IV group B refractory category, predominantly presenting with lung infiltration and concurrent multiorgan involvement of the bone marrow and adrenal glands is described herein.

**Interventions:**

The disease was controlled only after timely adjustment to a gemcitabine combined with oxaliplatin + PD-1 inhibitor chemotherapy regimen.

**Outcomes:**

The patient now remains clinically stable with adequate oral intake and satisfactory sleep quality.

**Lessons:**

Clinicians should broaden diagnostic considerations and pay greater attention to heterogeneous tumors. For patients presenting with persistent recurrent fever, pulmonary consolidation lesions, and poor response to anti-infective therapy, prompt percutaneous lung biopsy should be performed to obtain pathological confirmation.

## 1. Introduction

As a rare type of non-Hodgkin lymphoma, extranodal NK/T-cell lymphoma (ENKTCL) is highly aggressive and originates from NK cells and T cells. The nasal-type extranodal NK/T-cell lymphoma (ENKTCL-NT) primarily affects the nasal and nasopharyngeal regions, with common initial symptoms including nasal obstruction, rhinorrhea, or epistaxis, which gradually progress to edema, ulceration, perforation, and involvement of the upper respiratory tract and midface.^[1]^ According to the ENKTCL prognostic assessment model Ann Arbor staging system, most patients are classified as stage I/II, with limited dissemination to sites such as skin, lymph nodes, or central nervous system. Patients with stage III and IV disease have extremely poor prognosis, characterized by rapid progression and a 5-year survival rate below 30% after treatment.^[2]^ Regarding treatment, concurrent or sequential chemoradiotherapy remains the standard approach for stage I/II disease, while systemic chemotherapy is required for stage III/IV nasal-type and disseminated lymphomas.^[3]^

## 2. Case presentation

A 45-year-old man was referred to our hospital on January 4, 2022, because of “fever accompanied by cough and sputum production for over half a month.” The patient developed fever (peak temperature 39°C) with cough and white, copious sputum half a month ago after catching a cold. After showing poor response to intravenous antibiotic therapy administered at a community hospital, the patient was transferred to our hospital for further management.Chest CT findings revealed: bilateral pulmonary infiltrates; multiple small mediastinal lymph nodes. Auscultation of both lungs revealed decreased breath sounds with audible moistrales.

The laboratory test results obtained in our department were as follows: hs-CRP 15 mg/L, WBC count 5.2 × 10⁹/L, platelets 122 × 10⁹/L (↓), neutrophils 60.10%, lymphocytes 19.90%, monocytes 18.50%. PT-INR 1.17, AST 59 U/L (↑), LDH 397 U/L (↑), glucose 6.69 mmol/L (↑), uric acid 154 μmol/L (↓), albumin 34.2 g/L (↓), AST 52 U/L (↑), GGT 60 U/L (↑), LDH 416 U/L (↑), prealbumin 194.3 mg/L (↓). Tumor markers (lung): neuron-specific enolase 18.41 ng/mL (↑), ferritin 726.20 ng/mL (↑). Total IgE antibody 120.89 U/mL (↑); T-cell subset, CD4+/CD8 + ratio 2.94. Four-item infectious disease panel, G-test, GM-test, ANCA, ANA, procalcitonin, respiratory 9-pathogen panel, cryptococcal capsular antigen test, adenovirus antibody, and tuberculosis-specific T-cell assays were normal. Midstream urine culture and sputum culture were negative. Acid-fast bacilli smear, fungal smear, general bacterial smear, bilateral aerobic, and anaerobic blood cultures were all negative.

**Figure 1.**
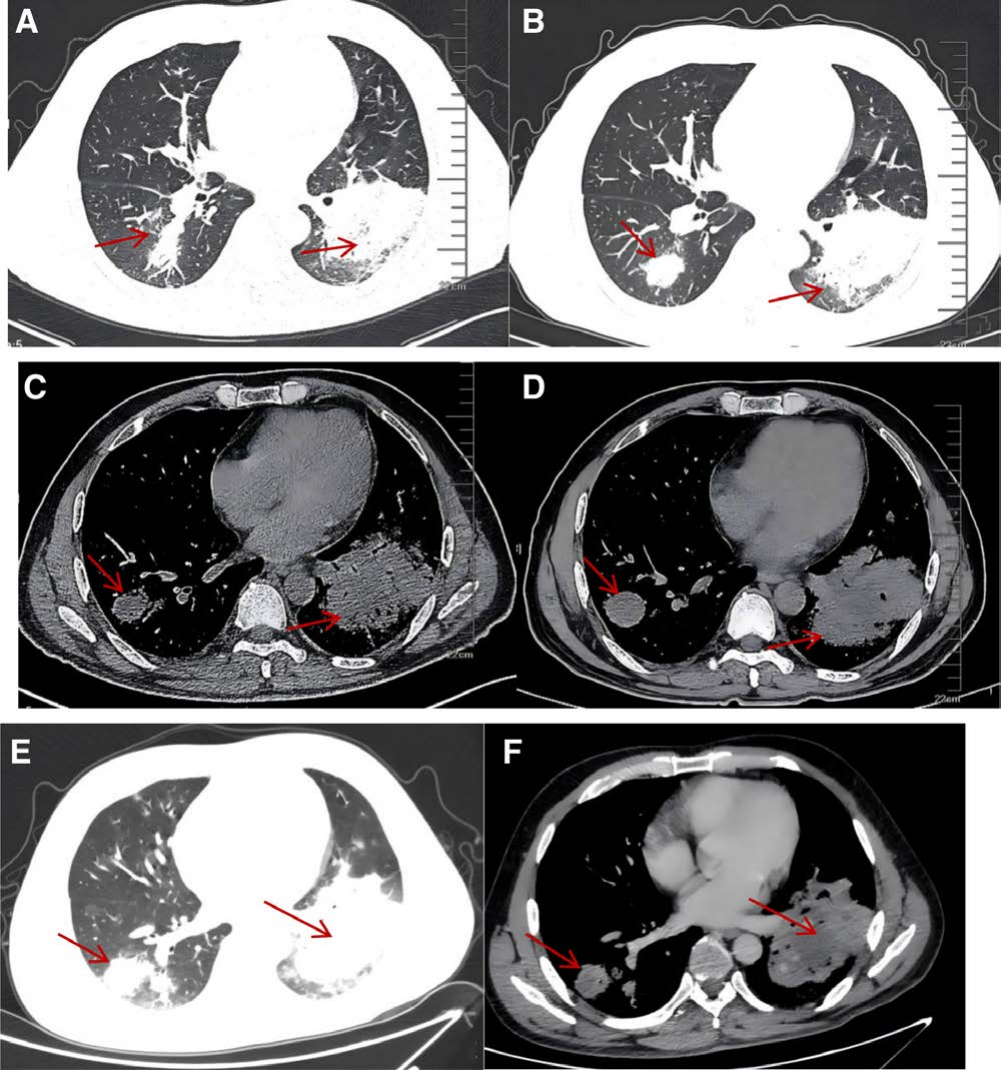
Chest imaging data. (A, C) January 3, 2022: multiple nodular, patchy high-density shadows, and small patchy ground-glass opacities were seen in both lungs. (B, D) January 11, 2022: multiple nodular, patchy high-density shadows, and small patchy ground-glass opacities were observed in both lungs, with the left lower lobe being more prominent; there was a significant progression compared to the previous examination (indicated by the red arrows). (E, F) January 17, 2022: multiple nodular, patchy high-density shadows, and small patchy ground-glass opacities were observed in both lungs, especially in the left lower lobe. Additionally, there were patchy and mass-like low-density areas. After enhancement, moderate enhancement was seen at the margins, while the central areas remained non-enhanced.

**Figure 2.**
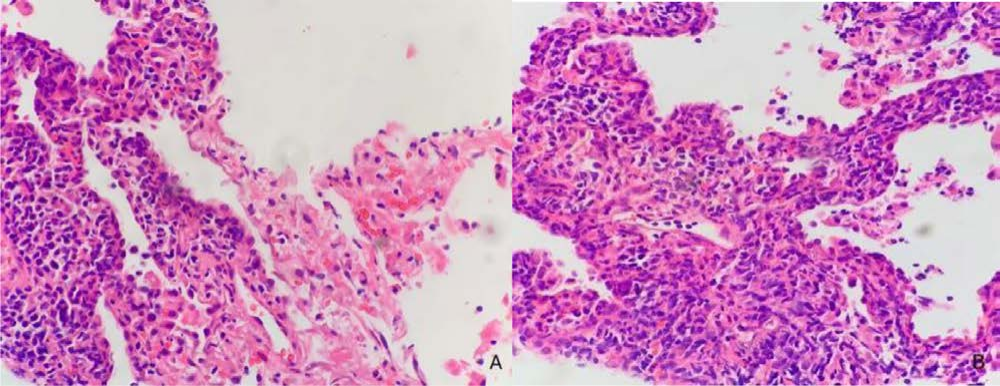
(A, B) January 18, 2022: a percutaneous lung biopsy was performed. (A) Following hematoxylin and eosin staining, infiltration of aberrant atypical lymphocytes into the alveolar septa accompanied by prominent necrosis was observed (200×). (B) Microscopic examination revealed polymorphic lymphocyte infiltration centered around blood vessels. Hematoxylin and eosin staining demonstrated tumor cells of intermediate size with irregular or convoluted nuclei, condensed chromatin, scant cytoplasm, and occasional small nucleoli (400×).

**Figure 3.**
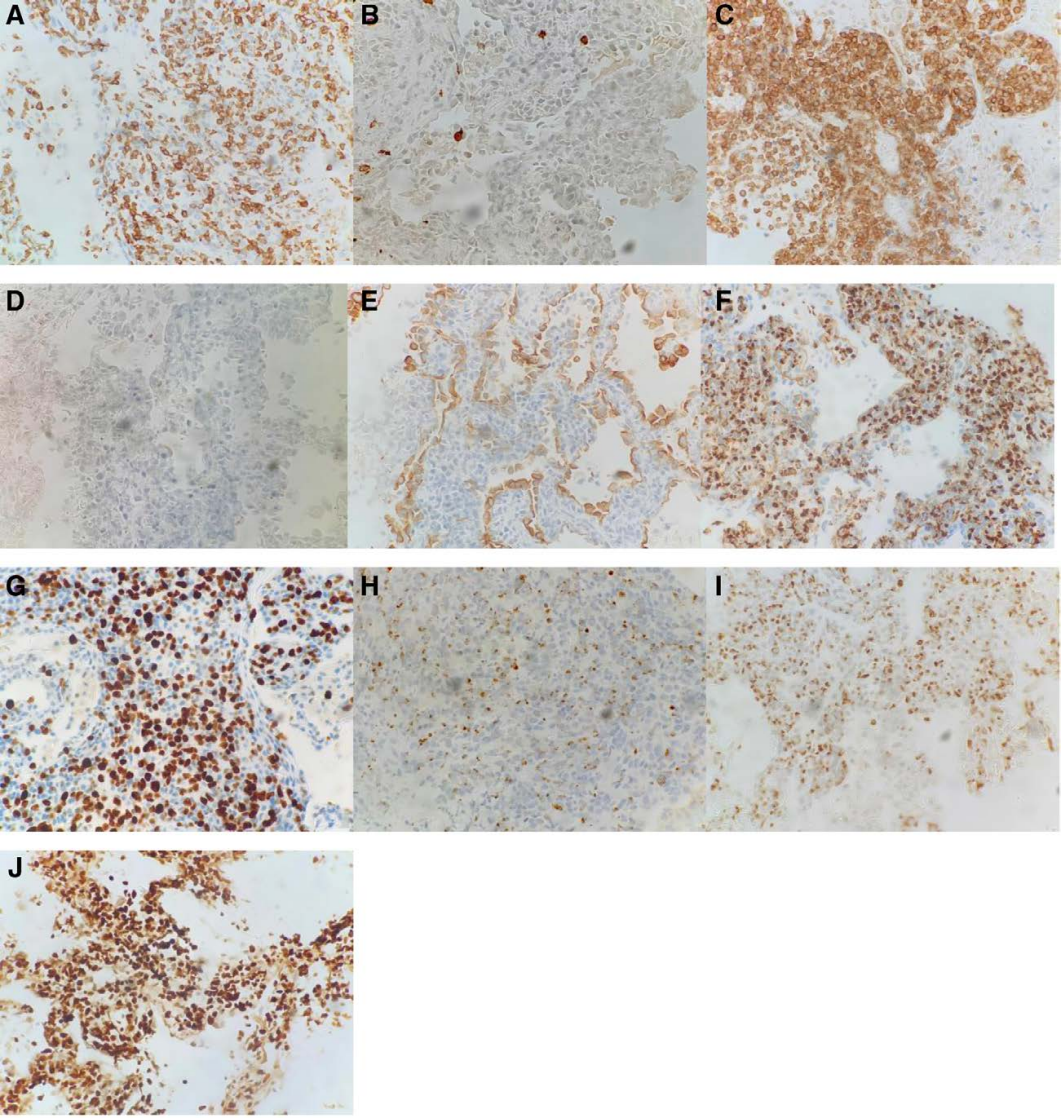
Immunohistochemistry of lung issue sample. (A–I) Immunohistochemical results of lung histopathological examination (200×): CD3 (+); CD20 (-); CD56 (+); CD79a (-); CK (-); GranB (+); Ki67: 80%; Perforin (+); TIA1 (+). (J) Biopsy lung molecular pathology results: EBER in situ hybridization (+).

After admission, the patient developed intermittent high fever, reaching 39.1°C, without chills and chills, coughing and expectoration, the amount of sputum was not much, mainly dry cough. Moxifloxacin 0.4 g once daily, piperacillin–tazobactam 4.5 g twice daily for anti-infection, ambroxol hydrochloride 2 mL intravenous injection twice daily for sputum resolution, Compound Paracetamol tablets as needed for fever and methylprednisolone 40 mg once daily to relieve toxemia symptoms. After 2 weeks of treatment, the patient showed no clinical improvement. Follow-up chest CT scans on January 11, 2022, and contrast-enhanced CT on January 17, 2022, revealed significant progression of pulmonary lesions compared to previous imaging (Fig. 1). On January 18, 2022, a percutaneous lung biopsy was performed. Next-generation sequencing of lung tissue detected 268 Epstein–Barr virus (EBV) viral sequences. Histopathological examination showed atypical lymphocyte infiltration in alveolar septa with necrosis (Fig. 2). Based on clinical findings, immunohistochemistry, and Epstein–Barr virus-encoded small RNA (EBER) testing, the diagnosis was consistent with NK/T-cell lymphoma, nasal type, intermediate to high aggressiveness. Molecular pathology results were as follows: EBER in situ hybridization (+); immunohistochemistry results: CD56 (+), CD3 (+), Perforin (+), Granzyme B (+), TIA1 (+), Ki-67 (80%), CD20 (-), CD79a (-), CK (-). (Fig. 3). EBV DNA, 2.04 × 10³ copies/mL; EBV early antigen IgG (+). A PET-CT scan performed on January 26, 2022, revealed the following findings: multiple scattered patchy, flocculent, and nodular opacities in both lungs and subpleural regions with bronchial penetration, ill-defined margins, and increased fluoro-deoxy-glucose (FDG) uptake; increased FDG uptake in the right side of the L4 vertebral body and local vertebral arch, compatible with NK/T-cell lymphoma pulmonary infiltration and bone marrow infiltration in the context of clinical history; slightly thickened left adrenal gland with increased FDG uptake, suggestive of lymphoma infiltration.

After being diagnosed in our hospital, the patient was transferred to the hematology department of an external hospital for further management on January 29, 2022. Bone marrow puncture and biopsy: bone marrow routine: erythroid, granular, megakaryon hyperplasia active, platelet scattered in small clusters can be seen. Bone marrow pathology: no heterogeneous lymphocyte was found. The peaspartase + methotrexate + etoposide + dexamethasone (PMED) regimen began on February 3, 2022. The specific regimen was as follows: methotrexate 3600 mg d1, dexamethasone 40 mg d2–4, etoposide 0.12 g d2–4, peaspartic enzyme 3750 U d2, supplemented with hydration and alkaline treatment, liver and stomach protection, diuretic treatment, etc. On March 4, 2022, the second cycle of PMED regimen was started: methotrexate 3600 mg d1, dexamethasone 40 mg d1–4, etoposide 0.12 g d2–4, peaspartase 3750 U d2. The patient still had recurrent fever and pneumothorax. Symptomatic treatment was given. The disease was poorly controlled. On March 24, 2022, replacement regimen gemcitabine combined with oxaliplatin (Gemox) + PD-1: gemcitabine 1.7 g d1, oxaliplatin 170 mg d1. The patient still had recurrent fever and poor disease control. 0.1 g etoposide was added on April 2 and April 5, 2022, respectively. On April 7, 2022, Cedarbenamine 20 mg biw was administered orally, and on April 8, 2022, Tirellizumab 200 mg was added to improve her condition. Follow-up patients were continuously diagnosed and treated, and the 8th course of chemotherapy was completed on September 3, 2022 with Gemox + PD-1: gemcitabine 1.7 g d1, oxaliplatin 170 mg d1, ralizumab 200 mg d2. From November 8, 2022, the 1st cycle was maintained with 200 mg of relizumab.

On January 4, 2023, post-treatment PET-CT imaging for lymphoma demonstrated: reduction of patchy and nodular opacities previously scattered beneath the pleura and in both lungs; no enlarged lymph nodes in the cervical, thoracic, or abdominal regions; normal spleen size with no abnormal FDG avidity; absence of abnormal FDG-avid foci in the bone marrow. Imaging findings indicate excellent treatment response, consistent with complete metabolic response. The linear opacity in the left lower lobe has decreased compared with previous imaging, suggesting resolution of chronic inflammation. Left adrenal gland shows normal morphology, density, and no increased FDG metabolism. The monotherapy maintenance therapy of PD-1 was discontinued after 7 courses of treatment were completed on November 30, 2023. Laboratory tests on November 30, 2023, revealed as follows: WBC 5.93 × 10⁹/L, neutrophils 3.43 × 10⁹/L, hemoglobin 162 g/L, platelets 118 × 10⁹/L (↓), thrombin time 17.8 seconds (↑), specific gravity 1.013 (↓), ALT 83.3 U/L (↑), AST 46.7 U/L (↑), retinol-binding protein 22.88 mg/L (↓), HDL 0.95 mmol/L (↓), LDL 2.38 mmol/L (↓). The patient achieved clinical improvement (Table 1).

**Table 1:** Clinical timeline.

Date	Event	Details
December 15, 2021	Symptom Onset	Developed fever (max 39°C) following exposure to cold. Antibiotic treatment at community hospital showed no significant improvement.
January 04, 2022	Hospital Admission	Admitted. Treatment focused on anti-infection, expectoration (phlegm reduction), and alleviation of toxemic symptoms.
January 11/17, 2022	Diagnostic Imaging	CT scan revealed significant progression of pulmonary lesions compared to prior imaging.
January 18, 2022	Diagnostic Procedure	Underwent percutaneous lung biopsy.
January 26, 2022	Diagnosis Confirmed	Diagnosed: Extranodal NK/T-Cell Lymphoma, Nasal Type (Stage IV B).
January 29, 2022	Hematology Referral & Procedure	Referred to hematology department of external hospital. Underwent bone marrow aspiration and biopsy.
February 03, 2022	First-Line Chemotherapy Initiated	Commenced PMED regimen: • Methotrexate 3600 mg, d1 • Dexamethasone 40 mg, d1–4 • Etoposide 0.12 g, d2–4 • Pegaspargase 3750 U, d2
March 24, 2022	Regimen Change due to Treatment Failure	Switched to Gemox + PD-1 (Tislelizumab) regimen due to suboptimal disease control.
April 8, 2022	Treatment Adjustment for Recurrent Fever	Recurrent fever. Addition of Tislelizumab 200 mg resulted in clinical improvement.
September 3, 2022	Continuation of Therapy	Completed Cycle 8 of Gemox + PD-1: • Gemcitabine 1.7 g, d1 • Oxaliplatin 170 mg, d1 • Tislelizumab 200 mg, d2.
November 8, 2022	Initiation of Maintenance Therapy	Commenced Cycle 1 of Maintenance Therapy with Tislelizumab 200 mg.
January 4, 2023	Treatment Response Assessment	Imaging showed favorable treatment response. Efficacy evaluated as Complete Metabolic Response (CMR).
November 30, 2023	Completion of Maintenance Therapy	Completed 7 cycles of PD-1 monotherapy maintenance (Tislelizumab). Treatment discontinued.

Gemox = gemcitabine combined with oxaliplatin, PMED = peaspartase + methotrexate + etoposide + dexamethasone.

The patient now remains clinically stable with adequate oral intake and satisfactory sleep quality. Despite residual chronic inflammation observed on follow-up chest CT, the patient reported significant improvement in his overall health status. He traveled abroad by plane last year. He expressed his gratitude to the medical workers who had helped him.

## 3. Discussion

ENKTCL-NT is an aggressive malignancy strongly associated with EBV infection, classified as a rare disease. Epidemiological studies have demonstrated a distinct geographic predominance of ENKTCL-NT, with higher incidence rates observed in Asia and specific regions of the Americas.^[1,4]^ Historically, this disease was diagnosed under various terminologies, including polymorphic reticulosis and lethal midline granuloma. Advances in cytogenetic and molecular biological research led to the formal introduction of the term “ENKTCL-NT” in 1996, which was subsequently incorporated into the 2001 World Health Organization classification of lymphomas and retained in the 2017 revised classification.

ENKTCL-NT primarily involves the nasal cavity and upper respiratory tract, frequently accompanied by systemic symptoms such as fever, night sweats, and weight loss. Extranasal involvement in sites such as the skin, soft tissues, or gastrointestinal tract occurs less commonly, while direct pulmonary parenchymal invasion is exceedingly rare. Bone marrow involvement is reported in 1.9% to 16% of patients during disease progression,^[5]^ with a high propensity for developing hemophagocytic syndrome. Patients with concurrent hemophagocytic syndrome exhibit significantly reduced survival rates and often present with high-grade fever, pancytopenia, coagulopathy, and histiocytic hyperplasia with hemophagocytosis. Histopathological hallmarks of ENKTCL-NT include tumor cell infiltration with extensive necrosis and angiocentric polymorphic lymphoid infiltration at the lesion site.^[6]^ This case report describes a clinically rare presentation of ENKTCL-NT with direct pulmonary involvement.

Inflammation, as a host defense mechanism, plays a dual role in malignancy. Pro-tumor inflammation contributes to tumor initiation, progression, and metastasis, and is recognized as a hallmark of cancer.^[7]^ Wang^[2]^ demonstrated that ENKTCL patients in intermediate- and high-risk groups with elevated platelet-to-lymphocyte ratio and increased lactate dehydrogenase levels exhibited poorer prognoses. While the pathogenesis of ENKTCL-NT remains incompletely elucidated, EBV infection has been established as a critical driver. Multiple clinical studies^[8–10]^ have confirmed the strong association between ENKTCL-NT and EBV, with reported EBV positivity rates approaching 100% in some cohorts. In this case, both pulmonary tissue and blood tests were positive for EBV.

Elevated EBV-DNA copy numbers are recognized as an adverse prognostic factor. Monitoring peripheral blood EBV-DNA load is essential for disease diagnosis and prognostic evaluation, particularly since patients with pretreatment EBV-DNA levels exceeding 600 copies/mL demonstrate significantly worse outcomes. A multicenter retrospective study by Shen et al^[11]^ investigating the prognostic value of EBV in ENKTCL revealed that after balancing intergroup differences using the inverse probability of treatment weighting method, patients with EBV-DNA-negative status at initial diagnosis had markedly superior survival compared to EBV-DNA-positive counterparts. Consequently, serial EBV-DNA monitoring is imperative to guide therapeutic strategies and prognostic assessment.

ENKTCL-NT lacks specific clinical manifestations and pulmonary imaging characteristics, with a highly complex pathological mechanism. In early disease stages, lymphocytic atypia is often insufficiently pronounced, and tumor-specific markers such as CD56 and EBER typically exhibit low expression levels. Nevertheless, histopathological examination combined with immunophenotyping remains the diagnostic gold standard.^[12]^ Clinical staging for ENKTCL employs the CA staging system and Prognostic Index for NK/T-cell Lymphoma-Epidemiology score. Observational studies^[13]^ indicate generally poor prognoses, with nasal-type patients demonstrating a 5-year overall survival rate of approximately 54%.

For treatment, sequential chemoradiotherapy is the primary approach for early-stage ENKTCL-NT, while advanced or refractory cases may require immunotherapy or hematopoietic stem cell transplantation. Clinical evidence^[14]^ supports the efficacy of the P-Gemox (pegaspargase, gemcitabine, and oxaliplatin) and MESA (methotrexate, etoposide, dexamethasone, and asparaginase) regimens in improving outcomes for elderly patients and those with extranasal involvement. Advances in understanding ENKTCL pathogenesis and immunotherapy have positioned PD-1/PD-L1 inhibitors as promising options for refractory/recurrent cases. CAI J et al^[15]^ reported significant efficacy in a cohort of 9 advanced ENKTCL patients treated with PD-1 inhibitors combined with P-Gemox, achieving complete remission in 6 patients after a median follow-up of 10.6 months.

In conclusion, primary pulmonary extranodal NK/T-cell lymphoma represents a rare clinical entity with high misdiagnosis rates. Clinicians must maintain a high index of suspicion for this heterogeneous malignancy. For patients presenting with persistent high fever, pulmonary consolidation, and poor response to antimicrobial therapy, early percutaneous lung biopsy is critical for definitive diagnosis. ENKTCL-NT with direct pulmonary involvement follows an aggressive clinical course and carries a dismal prognosis. Current research priorities focus not only on optimizing existing chemotherapeutic agents but also on developing novel targeted therapies for advanced and relapsed/refractory cases.

## Acknowledgments

We acknowledge the support received from Nanjing Traditional Chinese Medicine Youth Talent Training Program (ZYQ20035).

## Author contributions

**Investigation:** Hui Zhang.

**Resources:** Haibo Ge.

**Supervision:** Hui Zhang, Haibo Ge.

**Writing – original draft:** Hui Zhang, Jiaming Yin.

**Writing – review & editing:** Jiaming Yin.
